# Splenic stiffness does not predict esophageal varices in children with portal hypertension

**DOI:** 10.1002/jpn3.70247

**Published:** 2025-10-27

**Authors:** Margaux Jezequel, Mohamed El Fayoumi, Madeleine Aumar, Léa Tran, Clémence Saingier, Hélène Behal, Matthieu Antoine, Fréderic Gottrand

**Affiliations:** ^1^ Department of Pediatric Gastroenterology Hepatology and Nutrition CHU Lille, Univ. Lille Inserm U1286 ‐ INFINITE ‐ Institute for Translational Research in Inflammation Lille France; ^2^ Department of Pediatrics CHU Brest Brest France; ^3^ Department of Pediatric Radiology CHU Lille Univ. Lille Lille France; ^4^ Unité Statistique, Evaluation Economique et Data Management (SEED), Maison Régionale de la Recherche Clinique, CHU Lille Lille France

**Keywords:** child, clinical prediction rule, elastography, esophagogastroduodenoscopy, liver stiffness

## Abstract

**Objective:**

To investigate ultrasound parameters, particularly splenic stiffness, as predictors of pediatric esophageal varices.

**Methods:**

We included all children aged 0–19 years who underwent esophagogastroduodenoscopy, for variceal screening or surveillance, and abdominopelvic ultrasound with splenic elastography measurement. We also recorded biological parameters (platelets count, albumin) to determine a clinical prediction rule (CPR). Derivation and validation cohorts were defined according to measurement date. Receiver‐operating characteristic (ROC) statistics and sensitivity, specificity, positive predictive value, and negative predictive value for the optimal threshold value were calculated, and used to assess the performance of each parameter.

**Results:**

Eighty derivation cohort children and 58 validation cohort children formed the study sample. Cohort characteristics did not differ for age, sex, distribution of varices but differ for some etiologies, abdominal surgery, spleen size, splenic stiffness measurement (SSM), ascites, and the presence of spontaneous portosystemic shunts. In the derivation cohort, splenic stiffness was the best independent predictor of esophageal varices, with an area under the ROC curve (AUC) of 0.83, a sensitivity of 0.90, and specificity of 0.73 at a threshold of 22 kPa. In the validation cohort, SSM was no longer significantly associated with esophageal varices at endoscopy, had a lower sensitivity of 0.26, and no other threshold could be found. Liver stiffness measurement (LSM) and CPR had a correct predictive value (AUC 0.70 for LSM; 0.78 for CPR in the derivation cohort and 0.64 for LSM; 0.71 for CPR in the validation cohort) for esophageal varices.

**Conclusions:**

SSM cannot be used as a single parameter to predict esophageal varices. LSM and CPR despite their lower AUC appear to much more robust measures with consistent results across cohorts.

## Abstract (highlights)

**What is Known:**

- Esophagogastroduodenoscopy, the gold standard, is an invasive procedure for diagnosing pediatric esophageal varices.
- Abdominal ultrasound coupled with elastography allows measurement of the degree of hepatic and splenic fibrosis.
- Although splenic stiffness is considered a good predictor of pediatric esophageal varices, validation studies have not been performed.

**What is New:**

- No single ultrasound parameter accurately predicts pediatric esophageal varices.
- In this validation study, which is the first of its kind, splenic stiffness does not predict esophageal varices in children with portal hypertension.

## Graphical abstract

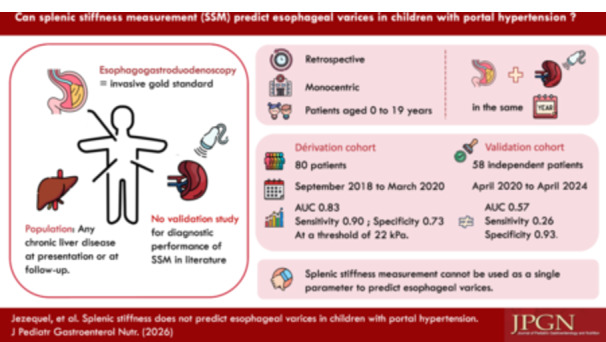

## 1 INTRODUCTION

While rare, pediatric portal hypertension (PH) remains the leading cause of upper gastrointestinal bleeding.^1^, ^2^ This bleeding originates from portosystemic shunts, particularly in esophageal varices,^3^ with a 5%–20% mortality rate.^4^ Early diagnosis of varices is thus a major challenge for primary prevention of bleeding. Although there is no formal consensus on primary prophylaxis to prevent variceal bleeding in children, screening endoscopy and prophylaxis treatment by ligation or sclerosis in cases of clinically significant varices (CSV) has been shown to be efficient.^5^, ^6^, ^7^, ^8^, ^9^ The gold standard for detecting esophageal varices is esophagogastroduodenoscopy (EGD), an invasive procedure requiring sedation. Noninvasive PH and esophageal varices predictors are therefore needed, to avoid unnecessary EGD.

Abdominal ultrasound (US), a noninvasive, nonirradiating examination, does not require sedation. It is highly informative regarding PH and its etiology^10^, ^11^ and can be coupled with elastography, a recent technological innovation for measuring degree of hepatic and splenic fibrosis. PH leads to histological spleen changes, with venous congestion followed by fibrosis, which increases elastography values.^12^

The Baveno consensus meeting recommended liver stiffness measurement (LSM) and splenic stiffness measurement (SSM) by transient elastography in adult patients with liver disease, instead of endoscopy to screen for PH^13^; a recent pediatric study shows good performance of the Baveno criteria (with LSM and not SSM) for screening CSV in biliary atresia.^14^ While several pediatric studies have suggested that splenic shear wave elastography (SWE) is a reliable predictor of esophageal varices,^10^, ^13^, ^15^, ^16^, ^17^, ^18^ no validation studies have yet been conducted.

Using pediatric cohorts including a validation sample, we aimed to evaluate the contribution of SSM in predicting the risk of esophageal varices. Other US measures and biological parameters were also assessed.

## 2 METHODS

### 2.1 Ethics statement

An information letter, with opposition form, were sent to patients' parents or legal guardians. The study was declared to the French Data Protection Authority (Commission Nationale Informatique and Libertés). All data were anonymized.

### 2.2 Study design

For this retrospective, single‐center study, children aged 0–19 years with endoscopy records at the Gastrointestinal Pediatric Department of the University Hospital of Lille were selected. Initial data collection included cases between September 2018 and March 2020, defined as the derivation cohort; the second data collection wave was between April 2020 and April 2024, defined as the validation cohort. We included all children who underwent EGD for esophageal varices and abdominopelvic US who had splenic elastography measurement within the same year. Indication for endoscopy was any chronic liver disease at presentation or at follow‐up if PH was suspected (US, splenomegaly, thrombocytopenia), or for surveillance.

Demographic, clinical, biological, and endoscopic data were collected from electronic medical records from the University Hospital of Lille. Clinical data included child age and sex, PH etiology, variceal bleeding history, and surgical history (e.g., Kasai procedure, liver transplantation, portal‐systemic shunt). Blood parameters were collected as close as possible to the endoscopy date, including alanine aminotransferase, aspartate aminotransferase, gamma‐glutamyl transpeptidase, total bilirubin, platelet count, prothrombin time, and serum albumin.

A clinical prediction rule (CPR) was calculated based on platelet count, spleen size Z‐score (SAZ), and albumin: [0.75 × platelet (10^9^/μL)/SAZ + 5] + 2.5 albumin (g/L). This clinical‐radiological score is used as a noninvasive predictor of the presence of esophageal varices.^19^

The EGD report detailed the presence and grade of esophageal varices from 1 to 3,^6^, ^7^, ^8^ red spot, mucosal friability, PH gastropathy, gastric varices, ulcers, and any associated endoscopic features. In the validation cohort, CSV and nonevolving varices were identified; CSV defined as grade 2 varices with red signs, grade 3 varices, or gastric varices along the cardia. Nonevolving varices were defined as stable or regressing varices compared with the previous endoscopy.

US data were collected from the dedicated radiology records of the University Hospital of Lille. All US scans were performed with the same machine, a Toshiba Aplio 500® (Toshiba America Medical Systems, Inc.) by a senior pediatric radiologist. Elastography measurements were performed according to good practice recommendations by SWE.^12^ At least five measurements were taken for each organ (liver and spleen), under apnea without Valsalva or indifferent breathing, >0.5 cm from the capsule and <5 cm deep. The following US measures were collected: liver size, liver stiffness, spleen size (SAZ), splenic stiffness, presence or absence of ascites, and presence or absence of spontaneous portosystemic shunts. SAZ is reported as standard deviations, compared with children of the same age and sex.^20^

When elastography measurements gave different values, we a priori decided to keep the highest one for the liver. Liver elastography can easily vary due to factors unrelated to PH (e.g., inflammation, viral infection), but the increase is greater in liver fibrosis. By using the upper range, the threshold obtained is less likely to be influenced by these small variations. We kept the lowest value for the spleen because we wanted to have the best sensitivity rather than specificity for detecting varices.

The first step of our analysis was to assess the diagnostic performance of the US measures and serum parameters according to EGD, considered the gold standard. The second step was to validate the thresholds identified in the first step in an independent sample, using the same methodology.

### 2.3 Statistical analysis

Continuous variables are expressed as the median (interquartile range [IQR]). Normality of distributions was assessed using histograms and the Shapiro–Wilk test. Categorical variables are expressed as count (percentage). The magnitudes of differences between the samples for demographic, radiologic, and endoscopic characteristics were assessed by calculating standardized differences. Absolute standardized differences >0.20 were interpreted as meaningful.

In the derivation cohort, the ability of US measures and CPR to diagnose esophageal varices was evaluated by receiver‐operating characteristic (ROC) curve analysis, by calculating the area under the ROC curve (AUC) with its 95% confidence interval (CI). The optimal cutoff value was determined by maximizing the Youden index. Sensitivity, specificity, positive predictive value (PPV), and negative predictive value (NPV) for the optimal threshold value were calculated with their 95% CI.

In the validation cohort, the diagnostic ability of the derivation cohort‐identified parameters was assessed, and the cutoffs identified, to estimate sensitivity, specificity, PPV, and NPV, and to compare their 95% CI between the two cohorts. In the validation cohort, US measures and CPR were compared between patients with and without CSV using the Mann–Whitney *U* test.

To assess relations between history of variceal bleeding and US parameters or CPR, the two cohorts were pooled due to the low frequency of the event. The association between variceal bleeding and US parameters or CPR was assessed using the Mann–Whitney *U* test.

Statistical testing was conducted at the two‐tailed α level of 0.05. Statistical analyses were performed using SAS software package version 9.4 (SAS Institute).

## 3 RESULTS

Eighty patients were included in the derivation cohort and 58 in the validation cohort. Their characteristics are reported in Table 1. The two samples were comparable on most clinical characteristics, except that history of surgery was more frequent in the validation cohort. The main etiology was biliary atresia in both groups with similar proportion although there was some difference in etiologies as 19% autoimmune hepatitis in the validation cohort versus 3.8% in the derivation cohort. US characteristics differed for spleen size, which was higher in the validation cohort. SSM was lower in the validation cohort. Ascites and spontaneous portosystemic shunts were more frequent in the validation cohort. The rate of esophageal varices was comparable, as was the grade distribution.

**Table 1:** Characteristics of the populations in the derivation and validation cohorts.

	Derivation cohort *n* = 80	Validation cohort *n* = 58	Standardized difference
Demographic data			
Male *n* (%)	46 (57.5)	28 (48.3)	0.19
Age (year)—median (IQ)	10.5 (4.0; 14.0)	9.20 (4.0; 14.3)	−0.05
History of upper GI hemorrhage *n* (%)	7 (8.8)	4 (6.9)	−0.07
Etiologies of portal hypertension			NA
Biliary atresia *n* (%)	22 (27.5)	19 (32.8)	
Portal vein obstruction *n* (%)	6 (7.5)	8 (13.8)	
Autoimmune hepatitis *n* (%)	3 (3.75)	11 (19)	
Congenital hepatic fibrosis *n* (%)	5 (6.25)	0 (0.0)	
Cystic fibrosis *n* (%)	10 (12.5)	2 (3.4)	
Parenteral nutrition associated liver disease *n* (%)	10 (12.5)	1 (1.7)	
Hepato‐renal polycystic *n* (%)	5 (6.25)	2 (3.4)	
Sclerosing cholangitis *n* (%)	3 (3.75)	1 (1.7)	
Wilson′s disease *n* (%)	0 (0.0)	3 (5.2)	
Alagille syndrome *n* (%)	0 (0.0)	3 (5.2)	
Others *n* (%)	16 (20.0)	8 (13.8)	
Surgery			**0.45**
No surgery *n* (%)	53 (66.3)	33 (56.9)	
Kasai *n* (%)	13 (16.3)	16 (27.6)	
Transplantation *n* (%)	8 (10)	5 (8.6)	
Porto‐systemic diversion *n* (%)	6 (7.5)	4 (6.9)	
Ultrasound data			
Liver size (mm)	103 (90; 120)	105 (94; 127)	0.20
Spleen size (SD)	2.9 (−0.1; 5.3)	4.3 (2.7; 7.2)	**0.48**
LSM (kPa)	12 (9; 17)	11 (9; 16)	−0.07
SSM (kPa)	25 (15; 38)	15 (12; 20)	**−0.80**
Ascites *n* (%)	2 (2.5)	4 (6.9)	**0.21**
Porto‐systemic shunts *n* (%)	9 (11.3)	19 (32.8)	**0.54**
Endoscopy			
Esophageal varices *n* (%)	40 (50.0)	31 (53.4)	0.07^a^
Grade 1	17 (42.5)	12/31 (38.7)	
Grade 2	22 (55.0)	18/31 (58.1)	
Grade 3	1 (2.5)	1/31 (3.2)	
PHG *n* (%)	33 (41.3)	25 (43.1)	0.04
Biology			
ALT (IU/L)	37 (23; 67)	51 (26; 117)	**0.31**
AST (IU/L)	50 (35; 76)	61 (40; 120)	**0.32**
GGT (IU/L)	26 (14; 56)	59 (20; 156)	**0.53**
Bilirubin (mg/L)	7 (3; 17)	7 (3; 19)	0.08
Platelet count (G/L)	151 (55; 276)	138 (96; 231)	−0.05
PT (%)	81 (64; 90)	82 (73; 93)	**0.22**
Albumin (G/L)	40 (36; 43)	39 (37; 43)	−0.01
CPR	117.7 (99.9; 136.2)	112.5 (101.0; 122.0)	**−0.26**

*Note*: Values are presented in terms of number (percentage) or median (IQR). Standardized differences were calculated between the two populations on the different characteristics. An absolute standardized difference >0.20 is considered as a more than ‘low’ difference.

Abbreviations: ALT, alanine aminotransferase; AST, aspartate aminotransferase; CPR, clinical prediction rule; GGT, gamma‐glutamyl‐transpeptidase; IQR, interquartile range; LSM, liver stiffness measurement; PHG, portal hypertensive gastropathy; PT, prothrombin time; SD, standard deviation; SSM, splenic stiffness measurement.

^a^ Calculated for the rate of esophageal varices (not according to the grade).

US parameters and CPR were first evaluated with the presence of esophageal varices in the derivation cohort (Table 2). Liver size had a low predictive value, with an AUC of 0.51. Thus, no cutoff could be identified. Spleen size and CPR had good predictive values, with AUCs of 0.77 and 0.78, respectively. LSM had a median predictive value, with an AUC of 0.70.

**Table 2:** Predictive value of ultrasound parameters and CPR to predict esophageal varices in the derivation cohort.

	AUC (95% CI)	Cutoff value	Sensitivity	Specificity	PPV	NPV
Liver size	0.51 (0.38–0.65)*	NA	NA	NA	NA	NA
Spleen size	0.77 (0.66–0.88)	≥3.58 (SD)	0.73 (0.58–0.87)	0.83 (0.70–0.95)	0.81 (0.67–0.94)	0.75 (0.62–0.88)
LSM	0.70 (0.58–0.82)	≥11.5 (kPa)	0.73 (0.58–0.87)	0.63 (0.47–0.78)	0.66 (0.51–0.80)	0.69 (0.54–0.85)
SSM	0.83 (0.73–0.93)	≥22 (kPa)	0.90 (0.80–1.00)	0.73 (0.58–0.87)	0.77 (0.64–0.89)	0.88 (0.76–0.99)
CPR	0.78 (0.66–0.89)	≤116.4	0.75 (0.61–0.90)	0.77 (0.63–0.91)	0.77 (0.63–0.91)	0.75 (0.60–0.90)

*Note*: Values in mean (SD). Spleen size in SD.

Abbreviations: 95% CI, 95% confidence interval; AUC, area under the ROC curve; CPR, clinical prediction rule; LSM, liver stiffness measurement; NA, not applicable; NPV, negative predictive value; PPV, positive predictive value; SD, standard deviation; SSM, splenic stiffness measurement.

**Figure 1:**
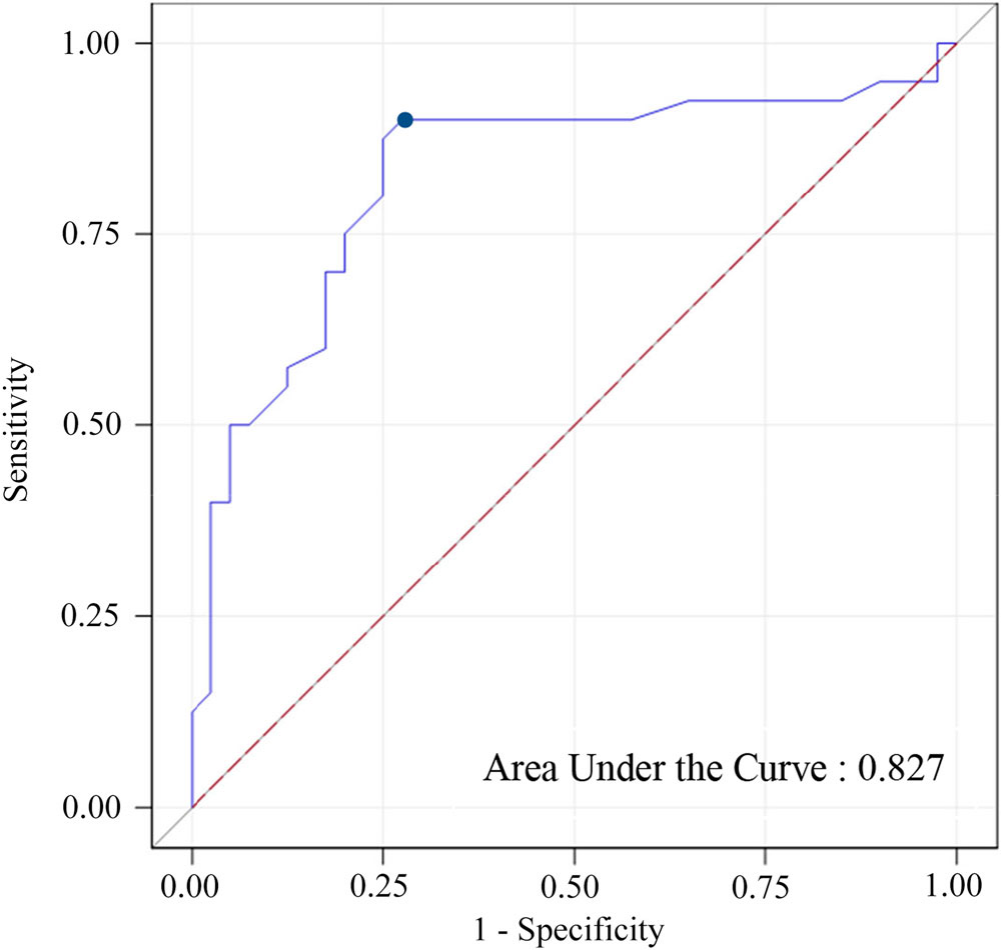
Receiver‐operator characteristic curve of splenic stiffness measurement for prediction of esophageal varices (22 kPa is represented by the blue dot).

The best predictive parameter to determine the presence or absence of esophageal varices at EGD was splenic stiffness, with an AUC of 0.83 (95% CI 0.73–0.93) (Figure 1). The value of 22 kPa was found to be the optimal threshold to predict esophageal varices. This cutoff had a sensitivity of 0.90 and a specificity of 0.72.

The combination of SAZ + LSM + SSM (AUC 0.85 [0.76–0.94]) or SSM + CPR (AUC 0.85 [0.76–0.94]) did not improve the predictive value compared with SSM alone (0.83 [0.73–0.93]) (Figure S1).

In the validation cohort, AUC was again calculated and found to be lower for all parameters than in the derivation cohort. Sensitivity, specificity, PPV, and NPV were calculated for each parameter and dichotomized according to the cutoff defined with the derivation cohort (Table 3).

**Table 3:** Diagnostic values of ultrasound parameters and CPR to predict esophageal varices in validation cohort.

	AUC (95% CI)	Cutoff	Sensitivity (95% CI)	Specificity (95% CI)	PPV (95% CI)	NPV (95% CI)
Spleen size	0.62 (0.47–0.78)	≥3.58 SD	0.67 (0.49−0.84)	0.46 (0.27−0.66)	0.59 (0.42−0.76)	0.55 (0.33−0.76)
LSM	0.64 (0.48–0.79)	≥11.5 kPa	0.60 (0.42−0.78)	0.58 (0.38−0.77)	0.62 (0.44−0.80)	0.56 (0.36−0.75)
SSM	0.57 (0.42–0.73)	≥22 kPa	0.26 (0.10−0.42)	0.93 (0.82−1.00)	0.80 (0.55−1.00)	0.52 (0.37−0.67)
CPR	0.71 (0.55–0.88)	≤116.4	0.82 (0.65−0.98)	0.64 (0.43−0.84)	0.69 (0.51−0.87)	0.78 (0.58−0.97)

*Note*: Values in mean (SD). Spleen size in SD.

Abbreviations: 95% CI, 95% confidence interval; AUC, area under the ROC curve; CPR, clinical prediction rule; LSM, liver stiffness measurement; NA, not applicable; NPV, negative predictive value; PPV, positive predictive value; SSM, splenic stiffness measurement.

Sensitivity for spleen size ≥3.58 SD was comparable between the two cohorts, with values of 0.67 in the validation cohort and 0.73 in the derivation cohort, but a lower specificity, PPV, and NPV in the validation cohort.

Sensitivity, specificity, PPV, and NPV of the liver stiffness threshold of 11.5 kPa were comparable between the derivation and validation cohorts, although slightly lower in the validation cohort.

Concerning CPR ≤116.4, sensitivity, specificity, PPV, and NPV were comparable between the cohorts.

The good diagnostic performance of splenic stiffness in the derivation cohort was not replicated in the validation cohort. Indeed, SSM was increased according to the threshold found in the derivation cohort in two patients presenting no esophageal varices, and was normal in 23 of 31 patients with esophageal varices (Figure S2).

The AUC of 0.57 (95% CI 0.42–0.73) was not significant. A splenic stiffness ≥22 kPa had very poor sensitivity (0.26) but very high specificity (0.93) in the validation cohort, comparable PPVs between the cohorts, and a lower NPV in the validation cohort.

To explore the reasons for the poor performance of splenic stiffness, we performed subanalyses controlling for several patient characteristics.

### 3.1 Nonprogressing PH

Eleven patients (19%) in the validation cohort had low splenic elastography (compared with the derivation cohort threshold) associated with esophageal varices, but these were stable or regressing compared with the previous endoscopy. When excluding the patients with nonevolving varices, diagnostic performance improved slightly but remained poor and nonsignificant, with an AUC of 0.63 (95% CI 0.46–0.79), sensitivity of 0.32, and specificity of 0.93 for the ≥22 kPa threshold in *n* = 47 patients.

### 3.2 Spontaneous portosystemic shunts

When excluding the 19 patients with a spontaneous portosystemic shunt, diagnostic performance of splenic stiffness did not improve, with an AUC of 0.60 (95% CI 0.42–0.78), sensitivity of 0.25, and specificity of 0.95 for the ≥22 kPa threshold in *n* = 39 patients. The distribution of grades of varices was also similar in the shunt and non‐shunt groups ( *χ*² = 2.81, *p* = 0.422).

### 3.3 Causes of PH

When excluding the nine patients with extra‐hepatic PH, the diagnostic performance of splenic stiffness did not improve, with a sensitivity of 0.20 and specificity of 0.92 for the ≤22 kPa threshold in *n* = 49 patients.

### 3.4 Role of previous liver transplantation or surgical shunt

When excluding the nine patients with liver transplantation and portosystemic diversion, the diagnostic performance of splenic stiffness was similar, with a sensitivity of 0.21 and specificity of 0.90 for the ≥22 kPa threshold in *n* = 49 patients.

### 3.5 Previous digestive bleeding or varices treatment

When excluding the 10 patients with a history of digestive bleeding or varices treatment, diagnostic performance improved slightly but remained poor and nonsignificant, with sensitivity of 0.32, and specificity of 0.91 for the ≥22 kPa threshold in *n* = 48 patients.

### 3.6 Time between EGD and US

When excluding the 24 patients with a delay >3 months between EGD and US, the diagnostic performance of splenic stiffness was similar, with a sensitivity of 0.25 and specificity of 0.93 for the ≥22 kPa threshold in *n* = 34 patients. Even when the interval was shortened to 7 days, the diagnostic performance remained poor, with a sensitivity of 0.17 and a specificity of 0.83 among 12 patients.

US measures with an AUC > 0.70 were compared according to the grade of esophageal varices in both cohorts (Figure S3). None of the parameters differed significantly between grades of esophageal varices.

In the validation cohort, no US measurement or CPR differed significantly between patients with CSV (grade 2 varices with reds signs or grade 3) and those without (Table S1).

## 4 DISCUSSION

Our cohort represents one of the largest samples using splenic elastography to predict pediatric esophageal varices, including 138 patients and is the first study to meet the quality standards of a validation study through rigorous methodology. However, results varied significantly between the two cohorts. The good diagnostic performance of SSM observed in the derivation cohort was not replicated in the validation cohort, where a high number of false negatives were recorded. Specifically, in the validation cohort, SSM failed to predict the presence of esophageal varices or CSV. When compared with the literature, several previous studies reported good sensitivity (0.70–0.75 for esophageal varices and 0.90–0.98 for CSV) and specificity (0.56–0.87 for esophageal varices and 0.57–0.80 for CSV), with a wide range of cutoff values (4.5–29 kPa).^13^, ^15^, ^16^, ^17^ The study by Vadlapudi et al. is relatively consistent with our results, showing that splenic elastography does not reliably predict CSV risk in children with extrahepatic PH.^18^ Unlike our study, none of the previous study replicated their finding in an independent sample. Without a proper validation study, there is a high risk of bias,^21^ and generalization to the wider population is not possible. However, the discrepancy between the two cohorts in our study prompted further investigation.

First, one consideration is the intrinsic variability of splenic elastography, which can differ depending on technique, US probe used, and factors such as obesity or ascites.^10^, ^22^ Previous studies have reported variable values and thresholds for splenic stiffness.^10^, ^11^, ^13^, ^15^, ^16^, ^17^, ^20^

Thus, a strength of our study is the use of the same US machine and probe for all participants, in accordance with best practice recommendations, minimizing external variation. However, ascites was significantly more frequent in the validation cohort (6.9% vs. 2.5%) which could have influenced measurements, although its overall prevalence remained low. BMI was not measured, so the impact of obesity could not be assessed.

There were no changes in the recruitment strategies or follow‐up between the two cohorts. The same team of clinicians, endoscopists and radiologists managed both groups. The criteria for endoscopy remained unchanged, namely any chronic liver disease at presentation or at follow‐up when PH was suspected (US, splenomegaly, thrombocytopenia), or for PH surveillance.

We cannot exclude the potential impact of the COVID‐19 pandemic, as the start of the pandemic coincided with the start of recruitment for the validation cohort. Operating room access was initially limited, but by the end of 2021, endoscopy access had returned to normal. Recruitment was extended until 2024 to mitigate any delays. Although delayed diagnoses might have led to more advanced PH and larger varices, this was not observed in our data.

However, as we gained experience and confidence with splenic elastometry, we probably used it more frequently and earlier in the disease course. As a result, the validation cohort likely included children with less severe PH compared to the derivation cohort. This also resulted in the recruitment of patients who had already undergone endoscopy without elastometry earlier in their follow‐up. This may have led to longer follow‐up times, with more patients who were not treatment‐naive and a higher number of children posttransplantation or with prior portosystemic shunts.

Indeed, the derivation cohort includes patients with non‐evolving esophageal varices and low elastography values (19%). Suggesting moderate PH insufficient to increase pre‐existing varices. Excluding these patients, however, did not significantly improve the ROC curve. Unfortunately, comparable data were not available for the derivation cohort.

Previous surgery, including liver transplantation and portosystemic derivation, may influence the relationship between splenic stiffness and esophageal varices. Yuldashew et al. showed that SSM decreased after portosystemic shunt surgery, but remains elevated compared to controls without PH.^23^ Goldschimidt et al. found decreased but persistently high SSM values post‐liver transplant^10^ Nevertheless, in our study, diagnostic performance remained poor even after excluding children in the validation cohort with prior surgery.

Then, spontaneous portosystemic shunts were approximately one‐third more frequent in the validation cohort. Although this may be coincidental, such shunts can reduce splenic congestion and fibrosis, potentially explaining lower elastography values despite the presence of varices. While excluding patients with shunts on US did not improve diagnostic performance, but it significantly reduces sample size and limiting statistical power.

The main etiology in both cohorts was biliary atresia, consistent with prior studies focusing solely on this population,^17^ Some etiologies varied (e.g., autoimmune hepatitis, cystic fibrosis) and etiology is known to influence splenic stiffness. For instance, it tends to be lower in cases of extrahepatic obstruction, as shown in the study by Upadhayay et al. where AUC was slightly lower, and cutoff values higher in these patients compared to those with chronic liver disease.^15^ However, in our study, excluding patients with extrahepatic causes did not improve performance, making etiology an unlikely explanation. The variation appears coincidental, and the inclusion of a broad population without exclusion enhances the generalizability of our findings to children with chronic liver disease.

A key limitation of our study is the low prevalence of high SSM values and high‐risk varices, which limited the distribution of values and statistical power. Previous studies used presence of CSV^15^, ^18^ or any varices^13^, ^17^ as their primary outcomes. Although identifying varices requiring treatment is clinically relevant, we lacked a sufficient number of such cases. Our protocol assessed patients at their first assessment with combined paired and stiffness measurement. The natural history of PH is to worsen over time, evaluating patients at baseline inherently limits the presence of advanced varices.

Another limitation is the retrospective design, which some US exams performed several weeks apart from EGD. In contrast, studies such as that by Upadhyay et al.^15^ performed both assessments on the same day. In our study, excluding patients with more than a 3‐month gap or 7‐day gap between US and EGD did not improve significantly diagnostic accuracy. No patient underwent elastometry immediately after endoscopy or bleeding and there were no intercurrent treatments or bleeding episodes between procedures.

Splenic elastography might be a good marker of PH but not necessarily the presence of esophageal varices, as other portosystemic pathways may develop. A prior adult study demonstrated that splenic stiffness correlates well with portal pressure but is limited in diagnosing esophageal varices.^24^

Due to differences in US machines, probes, and operators,^10^, ^22^ and patient history,^10^, ^23^ defining a universal SSM threshold in children remains challenging. However, variation within the same individual over time may offer a promising alternative. Future studies should investigate changes in splenic elastography over time to determine if a “delta” could better predict esophageal varices than fixed thresholds.

No single parameter or combination, appeared to be a reliable predictor of esophageal varices in our cohorts. Liver elastography and CPR had lower predictive value but consistent across the two cohorts. Previous studies showing contrasting results for liver elastography.^10^, ^14^, ^16^ For CPR, our AUC values were consistent with previous findings using a threshold of 116.^18^, ^25^ The Baveno criteria^14^ were not assessed here, as the study focused specifically on the performance of splenic stiffness.

## 5 CONCLUSION

Our study cautions against the use of SSM for predicting esophageal varices in real‐world settings, as its diagnostic value varies significantly depending on patient selection.

In contrast, LSM and CPR, despite having lower AUCs, appear to be more robust and yield more consistent results across the two cohorts.

In conclusion, at present, we cannot recommend the use of ultrasonographic parameters alone to predict the risk of esophageal varices in children with PH.

Further studies are needed to determine whether SSM could nonetheless be valuable for patient monitoring, based on changes over time rather than a single threshold value.

## CONFLICT OF INTEREST STATEMENT

The authors declare no conflict of interest.

## Supporting information

Supplemental Figure S1.

Supplemental Figure S2.

Supplemental Figure S3.

Supplemental Table S1 (2).

supmat.
