# Supplementary figures and images for: Splenic stiffness does not predict esophageal varices in children with portal hypertension
Source: J Pediatr Gastroenterol Nutr. 2025 Oct 27;82(1):156–64. doi: 10.1002/jpn3.70247 (PMC12780471; doi:10.1002/jpn3.70247)

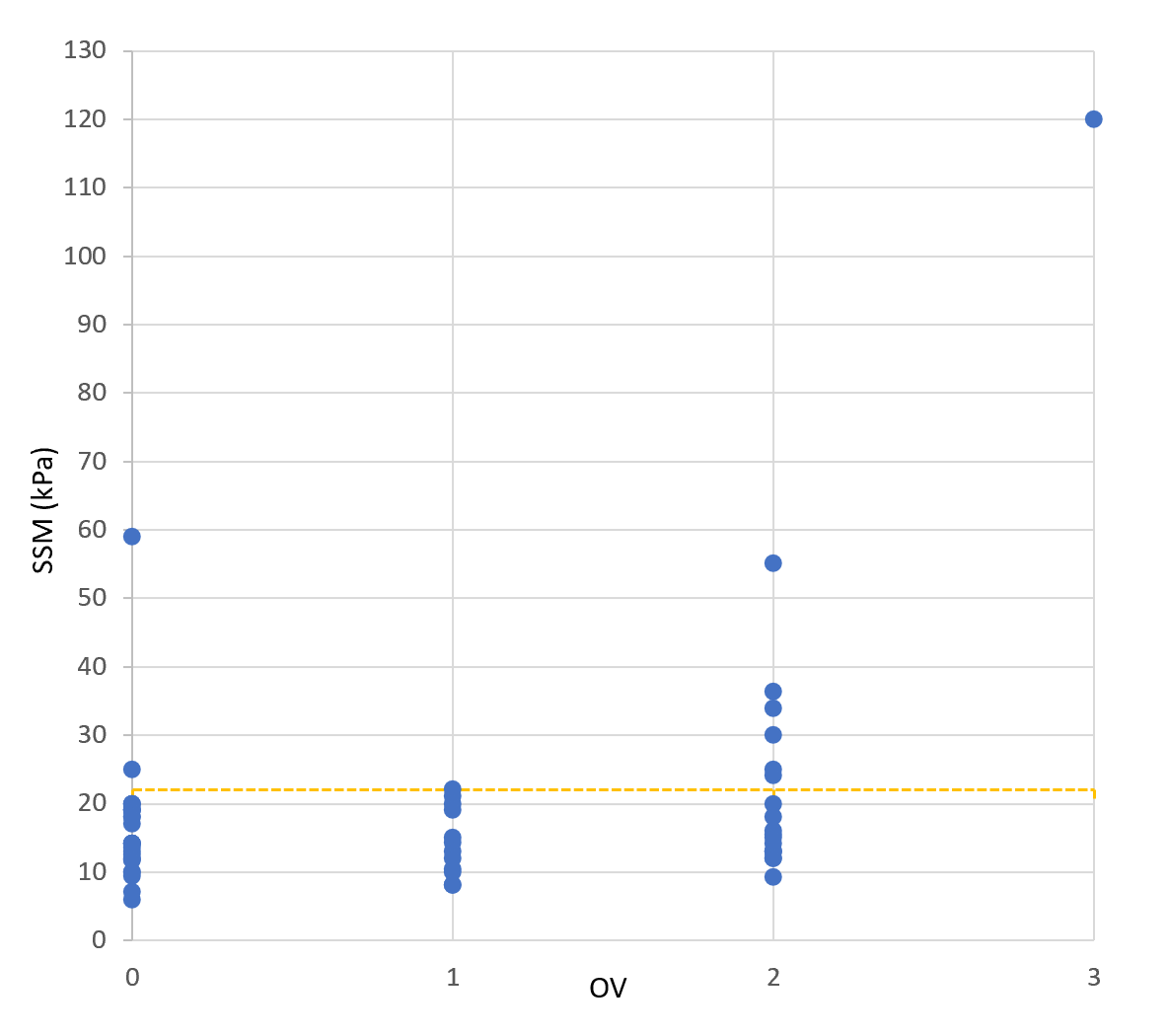

Supplement: Supplementary file 2 — Supplemental Figure S2. [file JPN3-82-156-s003.docx]

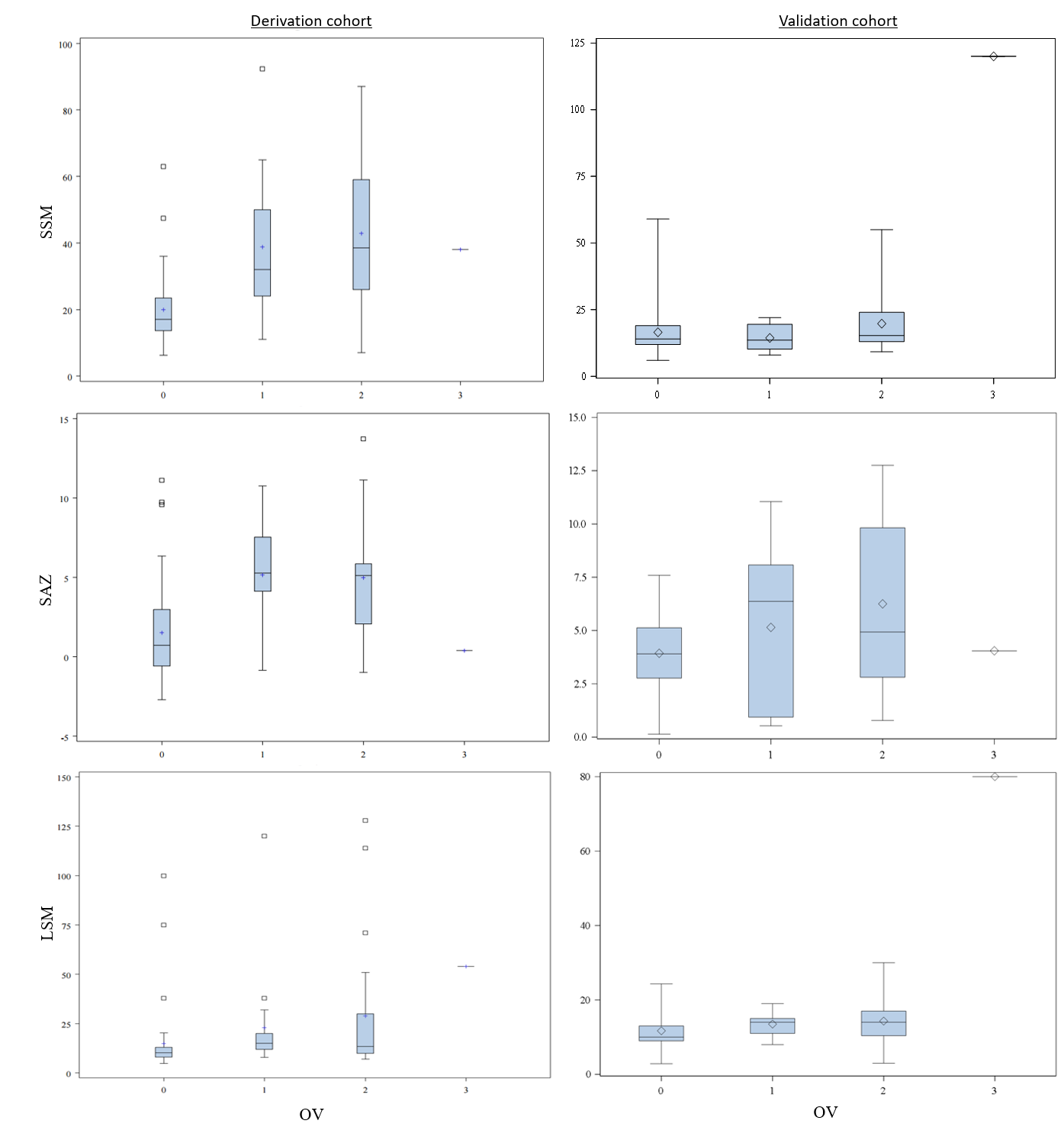

Supplement: Supplementary file 3 — Supplemental Figure S3. [file JPN3-82-156-s002.docx]
