# Supplementary material for: Splenic stiffness does not predict esophageal varices in children with portal hypertension
Source: J Pediatr Gastroenterol Nutr. 2025 Oct 27;82(1):156–64. doi: 10.1002/jpn3.70247 (PMC12780471; doi:10.1002/jpn3.70247)
Supplement: Supplementary file 4 — Supplemental Table S1 (2). [file JPN3-82-156-s004.docx]

|  | Non-clinically significant varices  N = 40 | Clinically significant varices  N = 18 | p-value |
| --- | --- | --- | --- |
| Liver size (mm) | 113 (98 – 128) | 100 (87 – 115) | 0.15 |
| Spleen size (mm) | 124 (100 – 150) | 137 (114 – 156) | 0.28 |
| SAZ (SD) | 3.9 (2.4 – 7.1) | 5.4 (4.0 – 7.2) | 0.078 |
| LSM (kPa) | 12 (9 – 17) | 11 (6 – 13) | 0.32 |
| SSM (kPa) | 15 (13 – 19) | 13 (12 – 24) | 0.69 |
| CPR | 116 (100 – 127) | 109 (102 – 116) | 0.34 |

**Supplemental Table S1.** Comparison of ultrasound parameters and CPR to predict clinically significant varices among patients in validation cohort.

95%CI= 95% Confidence Interval; PPV= Positive predictive value; NPV= Negative predictive value; SAZ= Spleen Size by Age; LSM = Liver stiffness measurement; SSM = Splenic stiffness measurement; CPR = clinical prediction rule;
