# Supplementary material for: Splenic stiffness does not predict esophageal varices in children with portal hypertension
Source: J Pediatr Gastroenterol Nutr. 2025 Oct 27;82(1):156–64. doi: 10.1002/jpn3.70247 (PMC12780471; doi:10.1002/jpn3.70247)
Supplement: Supplementary file 5 — supmat. [file JPN3-82-156-s001.docx]

Appropriate for the on-line supplemental digital content:

**Supplemental Figure S1.** Receiver-operator characteristic curves of splenic stiffness measurement (SSM) only (blue curve) and combined with spleen size Z-score and liver stiffness measurement (red curve at left) for prediction of esophageal varices or SSM combined with clinical prediction rule (red curve at right) for prediction of esophageal varices.

**Supplemental Figure S2.** Distribution of splenic stiffness measurement (SSM) by grade of varices in validation cohort. The yellow line represents the cutoff of 22▒kPa found in derivation cohort.

**Supplemental Figure S3.** Distribution of splenic stiffness measurement, spleen size Z-score in standard deviation and liver stiffness measurement by grade of esophageal varices in derivation and validation cohorts.

**Supplemental Table S1.** Comparison of ultrasound parameters and CPR to predict clinically significant varices among patients in validation cohort.

95%CI▒=▒95% Confidence Interval; PPV= Positive predictive value; NPV= Negative predictive value; SAZ= Spleen Size by Age; LSM = Liver stiffness measurement; SSM = Splenic stiffness measurement; CPR = clinical prediction rule;
