# Supplementary material for: Splenic stiffness does not predict esophageal varices in children with portal hypertension
Source: J Pediatr Gastroenterol Nutr. 2025 Oct 27;82(1):156–64. doi: 10.1002/jpn3.70247 (PMC12780471; doi:10.1002/jpn3.70247)
Supplement: Supplementary file 4 [file JPN3-82-156-s005.docx]

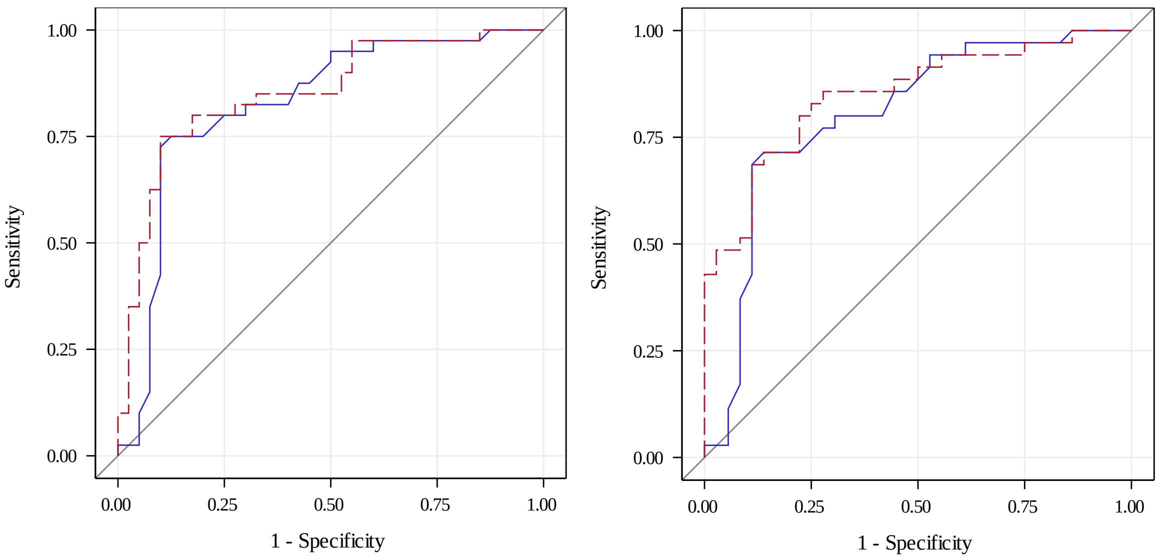

Sensitivity
1.00
0.75
0.50
0.25
0.00
0.00
0.25
0.50
0.75
1.00
1 - Specificity
Sensitivity
1.00
0.75
0.50
0.25
0.00
0.00
0.25
0.50
0.75
1.00
1 - Specificity
